# A Bayesian Meta-Analysis on Prevalence of Hepatitis B Virus Infection among Chinese Volunteer Blood Donors

**DOI:** 10.1371/journal.pone.0079203

**Published:** 2013-11-13

**Authors:** Guang-cong Liu, Guo-yuan Sui, Guang-ying Liu, Yang Zheng, Yan Deng, Yan-yan Gao, Lie Wang

**Affiliations:** 1 School of Public Health, China Medical University, Shenyang, Liaoning, PR China; 2 First Affiliated Hospital of Liaoning University of Traditional Chinese Medicine, Shenyang, Liaoning, PR China; The University of Tokyo, Japan

## Abstract

**Background:**

Although transfusion-transmitted infection of hepatitis B virus (HBV) threatens the blood safety of China, the nationwide circumstance of HBV infection among blood donors is still unclear.

**Objectives:**

To comprehensively estimate the prevalence of HBsAg positive and HBV occult infection (OBI) among Chinese volunteer blood donors through bayesian meta-analysis.

**Methods:**

We performed an electronic search in Pub-Med, Web of Knowledge, Medline, Wanfang Data and CNKI, complemented by a hand search of relevant reference lists. Two authors independently extracted data from the eligible studies. Then two bayesian random-effect meta-analyses were performed, followed by bayesian meta-regressions.

**Results:**

5957412 and 571227 donors were identified in HBsAg group and OBI group, respectively. The pooled prevalence of HBsAg group and OBI group among donors is 1.085% (95% credible interval [CI] 0.859%∼1.398%) and 0.094% (95% CI 0.0578%∼0.1655%). For HBsAg group, subgroup analysis shows the more developed area has a lower prevalence than the less developed area; meta-regression indicates there is a significant decreasing trend in HBsAg positive prevalence with sampling year (beta = −0.1202, 95% −0.2081∼−0.0312).

**Conclusion:**

Blood safety against HBV infection in China is suffering serious threats and the government should take effective measures to improve this situation.

## Introduction

HBV is a ubiquitous virus with global distribution and can cause acute and chronic liver diseases, which finally result in cirrhosis and hepatocellular carcinoma (HCC) [1]–[3]. Diseases caused by HBV infection roughly led to 1million death each year around the world, and it is estimated nearly 2 billion people have serological evidence of past or continuing HBV infectin [4].

As an endemic area of HBV infection, China has suffered great health and economic loss. Former studies have estimated that the number of HBsAg carriers in China could be 120 million [5], [6], which consist of about 10% of the whole population of mainland China in the early 1990s. Later studies reported that up to 20 million Chinese population sustained diseases caused by chronic hepatitis B, at the same time these diseases claimed 300,000 deaths annually in average [7], [8]. In 1992, a nationwide survey indicated that 9.75% of the Chinese populations are HBsAg positive [9].Consequently, Chinese Ministry of Health recommended routinely immunization against hepatitis B vaccine for infants since then. Although a follow-up survey carried out in 2006 indicated the prevalence had decreased, the prevalence was still as high as 7.18% [10].

Transfusion-transmitting of HBV was once rampant in China. Plasma economy which arose in the 1980s had developed rapidly and many illegal plasma collection centers were built for considerable profit. These centers usually pooled blood from different donors (even different types) together when filtering, and then transfused erythrocytes and haematoblasts back to paid donors [11]. This would undoubtedly cause serious epidemic of HBV, HIV and syphilis. One study carried out in Shanxi Province during the year 1990 reported a post-transfusion hepatitis B (PT-HB) incidence of 6.97% [12].

OBI can be defined as the lasting persistence of viral genomes in the liver tissue or serum of individuals negative for HbsAg [13]. Several studies reported that OBI could be transmitted by transfusion [14]–[17]. Unfortunately, OBI in Chinese volunteer blood donors has also been detected, adding an additional risk to blood safety of China.

To evaluate blood safety against HBV in China and help the government make informed decisions, dozens of studies aimed at investigating prevalence of HBV infection among Chinese volunteer donors were carried out [18]–[31]. However, the results varied substantially from one study to another, and divergence of this issue keep existing due to lack of nationwide investigation. But a nationwide investigation in a country consisting of over one billion people costs too much and requires a long time, Therefore the best way for us is to make use of current data and to present the comprehensive results by meta-analysis.

Two bayesian meta-analyses were performed. We hypothesized that: 1) prevalence of the normal HBV infection among Chinese volunteer blood donors was much higher than other countries since China has suffered epidemic of HBV infection, and economic level, education and sampling year should be covariates that lead to variation between studies; 2) Occult infection among donors was more serious than ambient countries of China; higher economic level yielded lower prevalence.

## Materials and Methods

### Study Design

Because HBV infections contain normal infection and occult infection, two analyses were performed independently in accordance with MOOSE [32]. We consider HBsAg positive as the indication of normal HBV infection. Articles were retrieved from Pub-Med, Web of Knowledge, Medline, Wanfang Data and CNKI. Relevant reference lists and potentially related studies were also screened. Language was limited to English and Chinese.

### Sample Size Calculation

Considering studies with too small sample size might not reveal the true prevalence, we calculate the minimum sample size that eligible studies should satisfy. To traditional calculation, we preferred the bayesian sample size calculation. Because in traditional approach, the standard deviation δ plays a vital role in determining the final sample size, but δ is usually unknown; besides, investigators need to determine the final sample size with other uncertainties such as the final observed data at planning stage [33], [34]. Thus bayesian sample size calculation in worst outcome criterion which ensures the desired coverage rate and interval length over all (or a subset of) possible datasets [35] was adopted, and we choose the worst outcome criterion (WOC) assuming the precision is unknown. The calculation was performed with help of R statistical package.

### Inclusion and Exclusion Criteria

Studies that met the flowing items are included: 1) studies reported sample size and the number of cases of HBsAg group or OBI group among Chinese blood donors 2) Considering that the prevalence of HBV infection among the whole Chinese population has significantly decreased attributed to the Blood Donation Law, eligible studies should collect samples after the law’s promulgation, here we choose after the year 2000 3) The sample size must be over our calculation result, and study location, sampling year, and reagents should be contained 4) donors included in the studies should pass the rapid test for HBsAg.

We eliminated studies with no information of reagents for detecting HBsAg. Samples that involved paid donors were also excluded. Publications that based on the same set of data were excluded except the paper with the most information.

### Search Strategy

An electronic search was performed up to 20^th^, October 2012. We screened Pub Med, Web of Knowledge, Medline and Wanfang Data using the terms “HBV infection”, “Blood donors”, “Prevalence” and “China”. In order to avoid publication bias, we also screened unpublished data from CNKI. The results were supplemented by a hand search of relevant references.

### Data Extraction

Two experienced authors extracted data from eligible studies independently. Information needs to be extracted was as follow: first author’s name, year of sampling, study location, sample size, gender ratio and cases of HBV infection. If one paper contains several sub-studies that were performed in different areas, we treated each sub-study as an independent one. Cases and sample sizes of that were sampled in the same year from different studies were added up to calculated annual data. Illiteracy rate, rate of employees in state owned units, unemployment and gross domestic product (GDP) per capita were obtained from Demographic Yearbook of China 2010. Latitude was identified using Google Earth. We classified each study location’s economic level according to whether Provincial GDP per capita exceeds 8000$ (Guangdong Province is also treated as developed area in respect of its economic status in China). All disagreements were settled by discussion till a consensus was reached.

### Statistical Analysis

Prevalence of the two events and corresponding 95% CIs were used to assess the blood safety against HBV infection. Compared to traditional meta-analysis, we preferred bayesian approach, which can take into account all sources of variations and reflect these variations in the pooled result [36], [37]. Consequently a hierarchical model which is most suitable for bayesian meta-analysis was adopted. We based both events on a binomial model since the prevalence ranged from 0 to 1.We consider a β distribution () as the best candidate for the non-informative distribution. Considering the numbers of cases and samples are often large, a and b were both expected to follow a uniform distribution [Uniform (0, 20000)]. Then subgroup analysis on potential factors was performed, following by bayesian meta-regression on sampling year, provincial illiteracy rate and gender ratio, rate of employee in state-owned units and unemployment if number of eligible studies is more than ten.

All bayesian analysis was performed with help of WINBUGS software. We entered bayesian models into WINBUGS and run 70000 Markov Chain Monte Carlo (MCMC) iterations, the first 20000 of which were used as burn-in. Then we checked the results’ convergence by calculating Geweke and Raftery&Lewis statistic using coda package of R. To check the stability of our result, we calculate the prevalence using another model. The bayesian models are presented in the Appendix S1.

The bayesian model will yield a posterior distribution based on the non-informative prior distribution. And the median of this posterior distribution with retrospective 95% CI were taken as the pooled prevalence. However, as to meta-regression, WINBUGS cannot yield a p value of the β coefficient, but we could judge its significance by whether its 95% CIs involved 0. All figures except the flow diagram were produced using R statistical package.

## Results

### Sample Size Calculation

The calculation result showed that sample size of included studies must be over 2152 in bayesian approach under WOC, with the parameters that length (fixed posterior credible interval length) = 0.2, level = 95%, n0 = 10, α = 2 (shape parameter of gamma distribution), β = 2 (scale parameter of gamma distribution), and WOS = 95%.

### Characteristics of Papers

**Figure 1:**
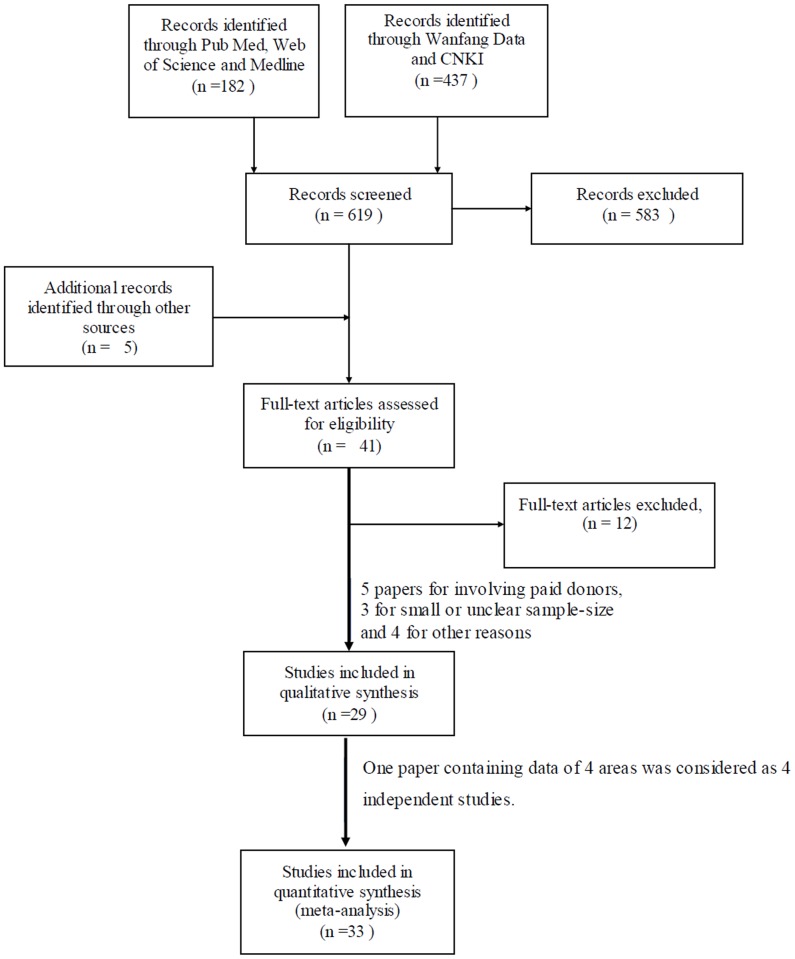
Flow diagram of study identification.

619 papers were identified in electronic search. 182 English papers were found, of which 26 were from Medline, 40 from Web of Science and 116 from Pub-Med. As to Chinese papers, 221 were indentified from Wangfang Data and 216 from CNKI. Five papers were added through hand search. Forty-one met our criterion after reviewing their abstracts. After full text reviewing, 5 papers were excluded for involving paid donors, 3 for small or unclear sample size and 4 for other reasons. Thus 29 papers were finally included, which 18 papers concerning normal infection and 10 concerning OBI, and one paper contains information for both two events. Another paper [38] containing data of 4 areas was considered as 4 independent studies. Hence 22 studies were collected for HBsAg group and 11 studies for OBI group among Chinese volunteer blood donors. Flow diagram of study identification was showed in figure 1.

Among the studies of HBsAg group gender ratios were available in 12 papers, but one [18] of them failed to report each gender’s prevalence. Fifteen studies reported number of cases and total donors year by year. Thus, meta-regressions on illiteracy rate, sampling year, latitude and gender ratio were all feasible. Unfortunately, little information about annual cases, gender ratio or number of cases of each genotype was found in studies of OBI group.

Among studies of HBsAg group, the highest prevalence was from a study that performed in Zhoukou [19], Henan Province, with a rate of 3.20%; and the lowest prevalence was 0.25% in Beijing City [20]. The largest sample size appeared in a study performed in Guangzhou with 2466834 donors [38] whereas the smallest sample was 4496 from Aba County, Sichuan Province [21]. To our surprise, the highest and lowest prevalence of OBI were both from studies performed in Sichuan [22], [23].

**Figure 2:**
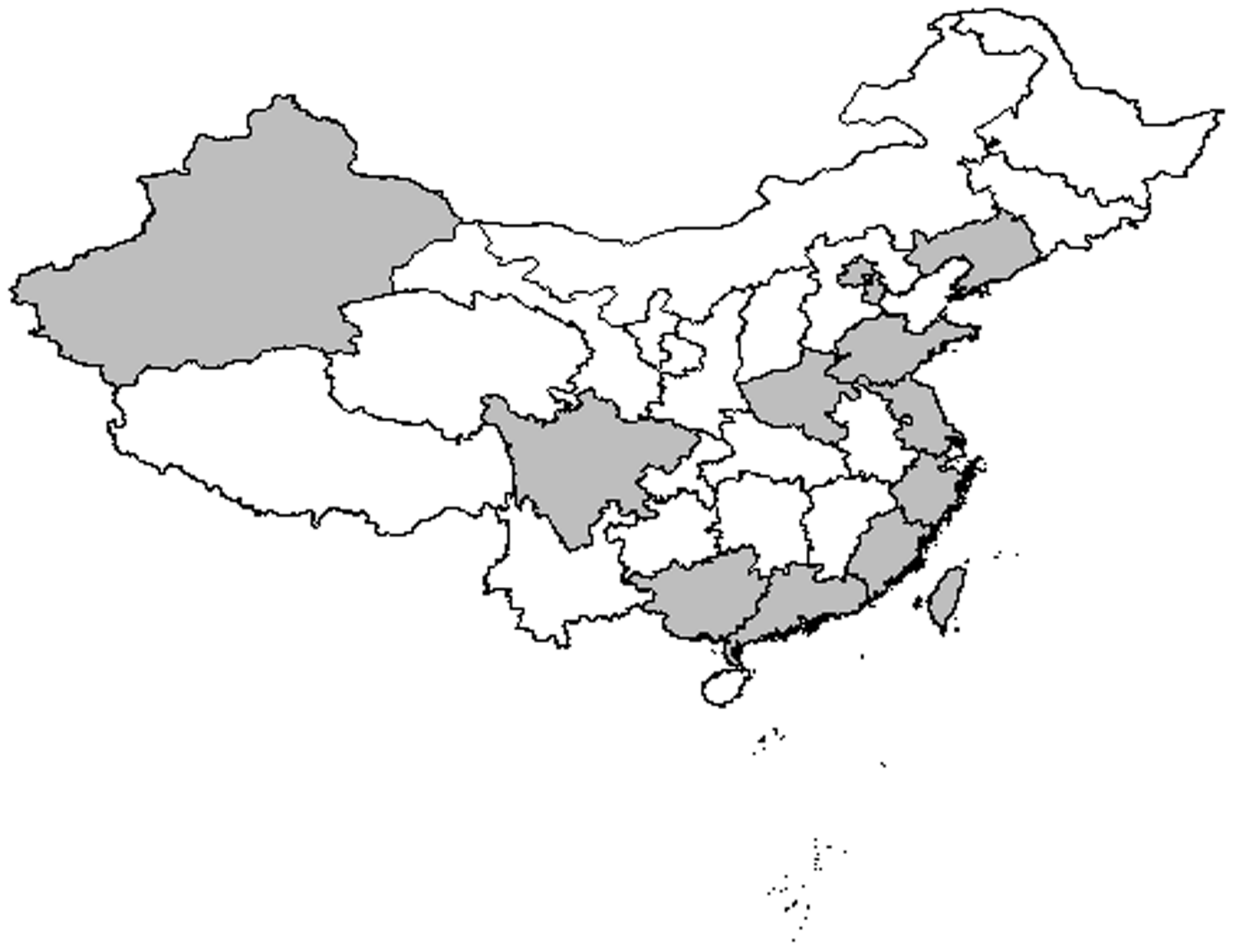
The 14 Provinces and automatic districts that covered in our study.

Study locations covered 14 provinces and autonomous districts of China, which is painted out in figure 2. A total of 5957412 donors and 53207 cases were included in HBsAg group and 571227 donors with 207 cases were presented in the OBI group. More characteristics of eligible studies in the HBsAg group and the OBI group were showed in table 1 and table 2, respectively.

**Table 1:** Characteristics of 22 eligible studies concerning the prevalence of HBsAg positive among Chinese volunteer blood donors.

Author	Sample size	cases	GR*	Prevalence	Location	Province	latitude	year	Economic	illiteracy	RESU*	GDP*
Wang MH	65263	161	1.75	0.25%	Beijing	BeiJing	39.54	2008	more	2.75	0.1479	12643
Li CQ	442259	1636	1.40	0.37%	Yancheng	Jiangsu	33.20	2000–2010	more	7.24	0.0615	9644
Zhou BQ	212692	821	NC	0.39%	Qingdao	Shandong	36.04	2008–2010	less	7.5	0.0785	7329
Feng J	158188	732	2.07	0.46%	Anyang	Henan	37.23	2007–2010	less	6.59	0.0640	4438
Li CQ	683704	3350	1.25	0.49%	Nanjing	Jiangsu	32.03	2000–2010	more	7.24	0.0615	9644
Chai JY	53034	297	NC	0.56%	Tanggu	Tianjin	39.01	2004–2007	more	3.07	0.1601	13193
Sun JZ	169091	1157	1.90	0.68%	Beihai	Guangxi	21.29	2005–2009	less	5.06	0.0688	3921
Deng WY	88010	630	NC	0.72%	Neijiang	Sichuan	29.35	2007–2011	less	3.63	0.0665	4046
Shang GF	176495	1409	NC	0.80%	Shenzhen	Guangdong	22.32	2001–2004	more	3.63	0.0690	7866
Quan Y	19581	158	NC	0.81%	Xiamen	Fujian	24.28	2007–2008	less	8.47	0.0699	7335
N Zaller	29784	264	1.51	0.89%	Urumiqi	Xinjiang	43.72	2003	less	3.4	0.2187	4658
Li CQ	2466834	22942	1.58	0.93%	Guangzhou	Guangdong	23.07	2000–2010	more	3.63	0.0690	7866
Li CQ	774570	9140	1.46	1.18%	Liaoning	Liaoning	41.50	2000–2010	less	3.2	0.1286	7859
Wang DL	39502	498	NC	1.26%	Chengdu	Sichuan	30.48	2004–2009	less	9.17	0.0665	4046
Xu DY	20417	267	NC	1.30%	pingdingshan	Henan	33.46	2010	less	6.59	0.0640	4438
Li QH	159721	1908	NC	1.31%	Jiangmen	Guangdong	22.34	2000–2004	more	7.81	0.0690	7866
Yang YL	20326	296	1.11	1.46%	Nanjing	Jiangsu	32.03	2008	more	7.24	0.0615	9644
Huang YD	161502	2385	1.48	1.48%	Zaozhuang	Shandong	34.48	2002–2009	less	7.5	0.0785	7329
Li FQ	4496	73	NC	1.62%	Aba	Sichuan	31.54	2006–2008	less	9.17	0.0665	4046
Zhu	43904	898	1.60	2.04%	Jinan	Shandong	39.39	2006	less	7.5	0.0785	7329
Yang	105879	2197	NC	2.59%	Jinan	Shandong	39.39	2002–2003	less	7.5	0.0785	7329
Li JQ	62160	1988	NC	3.20%	Zhoukou	Henan	33.38	2006–2010	less	6.59	0.0640	4438

GR: Gender ratio; RESU: Rates of Employees in State-owned Units; GDP: Gross Domestic Product per capita; NC: Not Clear.

**Table 2:** Characteristics of 11 eligible studies concerning the prevalence of OBI among Chinese volunteer blood donors.

author	Sample size	cases	prevalence	region	Province	latitude	genotype B	genotype C	year	economic
Chen	9023	17	0.1884%	Xiamen	Fujian	24.28	7	10	2009	developing
Zheng	165371	22	0.0133%	Shenzhen	Guangdong	22.32	NC	NC	2003–2009	developed
Li	199631	54	0.0270%	Guangzhou	Guangdong	23.07	NC	NC	2011	developed
Quan Y	19360	30	0.1550%	Xiamen	Fujian	24.28	11	19	2007–2008	developing
Su	10824	12	0.1109%	Taipei	Taiwan	25.05	NC	NC	2006–2008	developed
Yuen	13011	15	0.1153%	Hongkong	Hongkong	22.24	NC	NC	2005–2006	developed
Yuen	217595	67	0.0308%	Hongkong	Hongkong	22.24	NC	NC	2007–2009	developed
Zhang	135542	12	0.0089%	Chengdu	Sichuan	30.39	NC	NC	2007–2009	developing
Ji	9972	25	0.2507%	Lishui	Sichuan	28.28	13	12	2009–2010	developing
Liu	2972	5	0.1682%	Nanjing	Jiangsu	32.03	5	0	2009	developed
Ren	5521	5	0.0906%	Urumqi	Xinjiang	43.72	NC	NC	2008–2009	developing

NC: Not Clear.

### Result of Meta-analysis

The results of bayesian meta-analysis for normal infection and OBI are 1.085% (95% CI 0.859%∼1.398%) and 0.094% (95% CI 0.0578%∼0.1655%), respectively. In the multi-factor bayesian meta-regression, GDP per capita yield a significant result (β = −0.1222, 95% CI −0.2208∼−0.02846), but latitude, illiteracy, rate of employees in state-owned units and unemployment were detected not to be factors that lead to heterogeneity. However, sampling year was confirmed to be a significant covariate (β = −0.1202, 95% CI −0.2081∼−0.0313). Pooled prevalence of normal and occult infection using another model is 1.00% and 0.1096%.

### Subgroup Analysis

For normal infection, subgroup analysis on illiteracy rate showed that the lower illiteracy group (< = 5%) seemed to have a lower prevalence (prevalence = 0.751% 95% CI 0.564%∼1.01% versus prevalence = 1.236% 95% CI 0.926%∼1.683%). As to the economic level, there seemed a lower prevalence in the more developed areas than in the less developed area (prevalence = 0.739%, 95% CI 0.530%∼0.106% versus prevalence = 1.275%, 95% CI 0.975%∼0.170%). As for rate of employees in state-owned units, the higher rate yields a significantly lower prevalence (0.581%, 95% CI 0.401%∼0.857% versus 1.194%, 95% CI 0.937%∼1.552%).

For occult infection, more developed area also has a relatively lower prevalence than the less developed area (0.0475%, 95% CI 0.0275%∼0.0914% versus 0.1297%, 95% CI 0.0706%∼0.253%).

The convergences of the results’ posterior distribution in MCMC chain were stable and reliable by checking graph of chain. Geweke and Raftery&Lewis statistic confirmed our judgment. All but one value generated for the predictive prevalence was not between −2 and 2 in Geweke method. None of the stochastic nodes’ ratio was more than 5 in Raftery&Lewis method, indicating our results were reliable.

**Figure 3:**
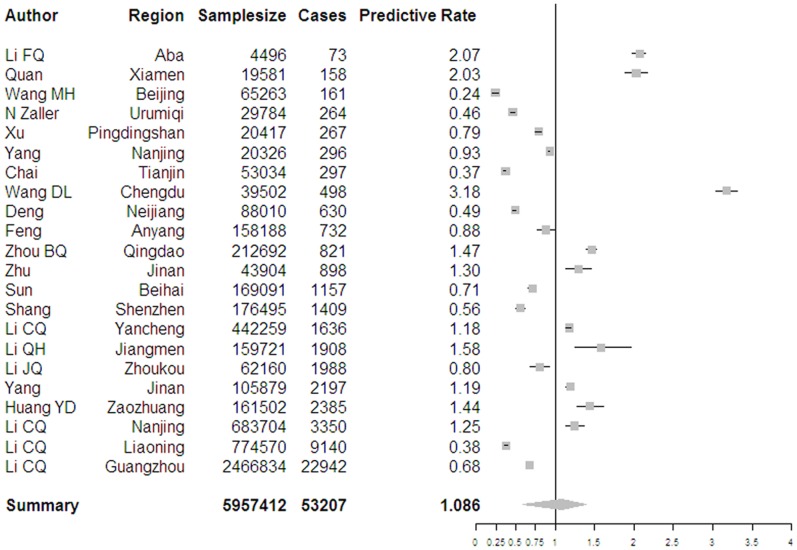
Forest plot of prevalence of normal infection among Chinese blood donors.

**Figure 4:**
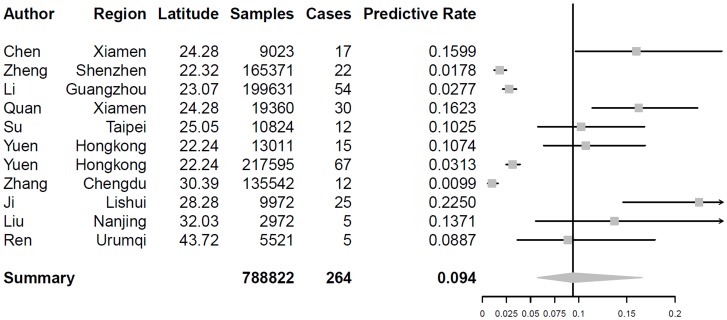
Forest plot of Occult HBV infection among Chinese volunteer blood donors.

**Figure 5:**
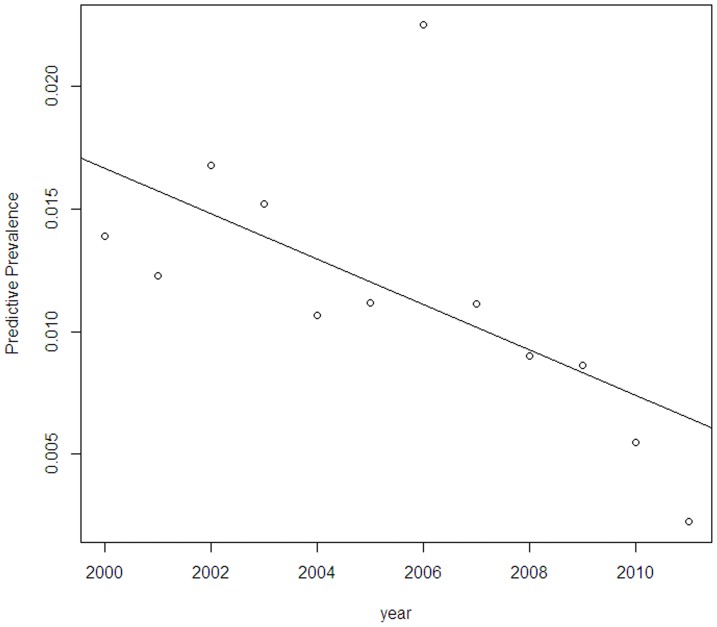
Bayesian meta-regression indicated sampling year negatively associated with the prevalence.

**Figure 6:**
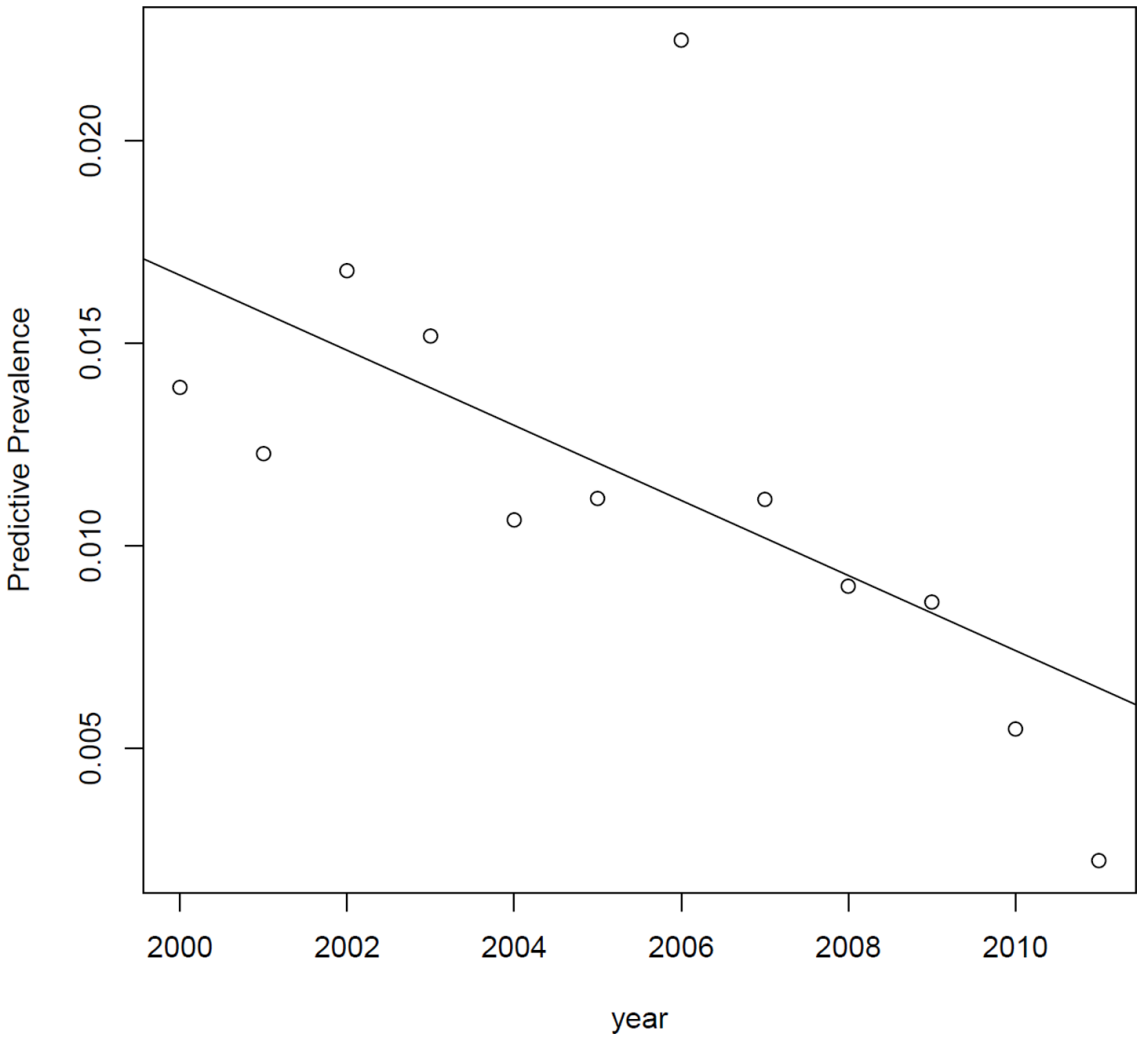
Bayesian meta-regression indicated GDP per capita negatively associated with the prevalence.

Forest plot of two events as well as graph of meta-regression was presented as figure 3, 4, 5 and 6.

## Discussion

Compared to the prevalence of normal infection among donors in China of 1.085%, those of other countries were obviously much lower: 0.12% in France [39], 0.23% in UAE [40], 0.004% in Britain, even the rates of most countries in Latin American are less than 0.5% [41].

The serious epidemic of HBV infection among Chinese population might account for the high prevalence of normal HBV infection among Chinese blood donors. The prevalence among Chinese whole population is 8.75% according to the first nationwide investigation in 1979 [31], although researchers considered this prevalence should be underestimated due to methodological deficiency. However, it kept increasing in the 1980s and the early 1990s, when paid plasma collection centers were prosperous in poor rural areas, many of which were illegal. Unlike whole-blood donation, plasma donation entails a shorter time to recover. Therefore paid donors always preferred this way. But unsafe measures such as reusing of non-sterile needles were often taken to pursue more profit. Some centers even pooled blood of the different types together to filter [24], [42]. Some centers did not do any pathogen detection before donation, which aggravated the spread of transfusion-transmitted diseases. Even if detections were done, their quality was usually poor [25]. Thus these tests might fail to identify high-risk donors including drug users.

On the other side, ascribing to traditional Chinese beliefs, many people persist that blood donation is harmful; other people still hold cautious opinion [43]. To make up the blood shortage, paid whole-blood donors are often required. These paid donors, typically came from rural areas in poverty, sold their blood just to make a living. Those who also donated plasma in illegal centers were often involved. Thus high-risk donors considerably increased the risk of transfusion-transmitting of HBV.

To make matters worse, tests in regular centers are not always guaranteed, either. Some blood centers only conducted one test of donors’ blood after rapid test [33]. Moreover, one study demonstrated that several reagents presented worrying sensitivity [26]. According to data from Chinese Society of Blood transfusion, the seropositive prevalence for HBsAg among Chinese whole-blood donors is 3.1% [26]. And the national immunization plan with hepatitis B vaccine did little help to blood safety against HBV infection during 2000 to 2010. Because volunteer donors under 18 is banned by blood donation law, while the first batch of infants immunized with vaccine were born in 1992 and they were under 18 until the year 2010. Thus, HBV infection among donors still poses a worrying problem to blood safety of China.

OBI among volunteer donors is still a potential threat to transfusion safety of China. Despite nucleic acid test (NAT) had been implemented in developed countries by the end of last century, NAT in China has just started in 2010 and is not widespread to date [44]. Moreover, an American study showed that NAT does not have sufficient sensitivity [45], which means OBI among donors may remain threatening even if NAT was adopted as a regular test.

This worrying condition poses a pressing and urgent problem which needs more attention from the Chinese government. Because many small hospitals in China cannot do pre-transfusion tests ascribing to technical limits, they have to ask blood centers to do the test for them. As mentioned above, some commonly used reagents in blood centers do not show satisfying sensitivity, and data implied that quality of tests is also worrying [26]. Therefore even a very low prevalence among donors may result in serious consequences.

Subgroup analysis for normal HBV infection revealed that the more developed areas seemed to have a lower prevalence. Generally speaking, more developed area often have a better health care system and input more on public health. With better awareness of self-protection and periodic medical examination, people in more developed areas are less possible to get infected. Patients who know their infection will not go to donate blood, either. As to developing areas, especially rural areas in poverty, most patients did not know their infection and the harm of contaminated blood, so they might donate blood as well.

Subgroup of higher rate of employees in State-owned units has a significantly lower prevalence. This may owe to different welfare and insurance level (including medical insurance). According to an unofficial survey performed in 2012, Corporation Employee Benefit Index (CEBI) in China is only 65.37; however, employees in state-owned units often get better welfare, and private units offered the worst welfare, where medical insurance were usually deprived [46]. As a result, employees in state-owned units, with higher level of welfare and insurance, will undoubtedly be less possible to get infected. In addition, the lower illiteracy group also gained a lower prevalence, which is believed to ascribe to the awareness of self-protection.

Meta-regression showed that sampling year is a significant covariate that leads to heterogeneity between different studies. Four reasons could account for it. First, the overall HBsAg positive prevalence among the whole population is decreasing year by year, so the prevalence among donors has decreased as well. Second, propaganda on blood donation is improved and more donors from low-risk group have been motivated. Third, the awareness of protection against HBV infection has been aroused, and more people realized their infection through medical test. Another reason is that with the rapid growth of normal blood centers, infected donors identified by regular test were banned from donating. Thus healthy repeated donors are accounting for more proportion. One study performed in Xi’an reported a significant lower rate of HBsAg positive in repeated donors [27]. Therefore a steadily decreasing trend appeared.

Prevalence of OBI among Chinese volunteer blood donors is also much higher than those of adjacent countries. OBI prevalence among Asian blood donors is 0.007% in Malaysia, 0.008% in Thailand and 0.004% in Singapore [28]. The huge discrepancies between prevalence may ascribe to 4 reasons as follow: 1) Appearance of OBI can be caused by abnormity of host immune response. Based on a higher prevalence of HBV infection, the OBI prevalence among donors may increase as well. 2) Compared to studies of other countries, studies in China have a relatively smaller sample size, so the results might be biased. 3) Cases of false positive can enlarge the discrepancy. 4) Some studies indicated that OBI appeared significantly more frequent in genotype C than in genotype B [29], [47]. One review demonstrated the incidence of genotype C increases with decreasing latitude [29]. In respect of included studies, few were performed in the north of China, that is, donors infected by HBV of genotype C account for more proportion, resulting in more possible for OBI.

The highest prevalence of normal infection is found in Henan Province. According to the Demographic Yearbook of China 2010, Henan Province had a population of over 100 million, and 67 million of them live in rural area. With an area of only 167 thousand square kilometers, the contradiction between people and land was the strongest one in China. An official report published in 2012 showed the income ratio of urban and rural residents is 3.3, and it is still increasing [48]. News on XINHUA website reported annual income per capita of nearly 11 million rural people in Henan Province was less than 2300 Yuan (about 369 US dollars) [49]. Living in such arduous situation, selling blood seemed to be the easiest way to make money, so plasma economy was once very rampant in Henan Province in the early 1990s. Moreover, lack of supervision might allow the contaminated blood being sent to public hospitals as blood collected from formal approaches, and risk of PT-HB significantly increased. However, no investigation on prevalence of HBsAg positive was carried out by officials of Henan Province. The government takes high prevalence as stains on their political achievements, and personal investigations are usually hampered.

Unfortunately, this result of normal infection should be underestimated. The latest nationwide investigation performed in 2006 indicated that the prevalence among the whole population is 7.18%, and rural population (7.3%), population in western area (8.3%), Zhuangs and Uigurs (8.2% and 13.4%, respectively) were at increased risk for HBV infection [11]. Another study reported that prevalence of HBsAg positive in Tibet was as high as 13% [30]. However, no studies aimed at exploring the condition of normal HBV infection among donors in high risk population that mentioned above were found. Because the areas with no studies are mostly less developed areas. Although HBV infection is endemic, staffs of blood center as well as researchers are not able to perform investigations due to conditional limits.

The deficiency of this paper must be pointed out. Studies included in our paper failed to cover all provinces of China, and few studies were performed in rural or poor areas, which may result in an underestimation of the true prevalence. Although sample size of each study is relatively enough, methodological quality of many studies, especially those written in Chinese, are not satisfying. As to the prevalence of OBI, given that only 11 studies meet our inclusion criteria, we cannot guarantee that it revealed the true prevalence. However, these deficiencies will not deny our contribution because we provided strong evidence confirming the severity of HBV infection among Chinese volunteer blood donors. These evidences can help with making informed decisions by the government of China.

## Conclusion

The high prevalence of HBV infection among volunteer blood donors in China calls for an effective testing system, and more potential donors in low risk should be motivated by promoting propagandas. Legislation should be made to protect medical welfare of employees in private companies, in order to decrease HBV infection. And better education of healthcare is needed. NAT should become a regular test for donors as soon as possible, and detection technology with more sensitivity is expected. Further, a nationwide investigation held by officials is required. Above all, the central government of China should input more into the construction of public health system in less developed area as an essential solution to improve blood safety against HBV infection.

## Supporting Information

Appendix S1 The statistical models of Bayesian meta-analysis and meta-regression.
(DOC)

Checklist S1 PRISMA 2009 Checklist.
(DOC)
