# Correction: A Bayesian Meta-Analysis on Prevalence of Hepatitis B Virus Infection among Chinese Volunteer Blood Donors

**DOI:** 10.1371/journal.pone.0105458

**Published:** 2014-08-06

**Authors:** The PLOS ONE Staff

Figure 6 is an accidental duplication of Figure 5. Please see the correct Figure 6 here.

**Figure 6:**
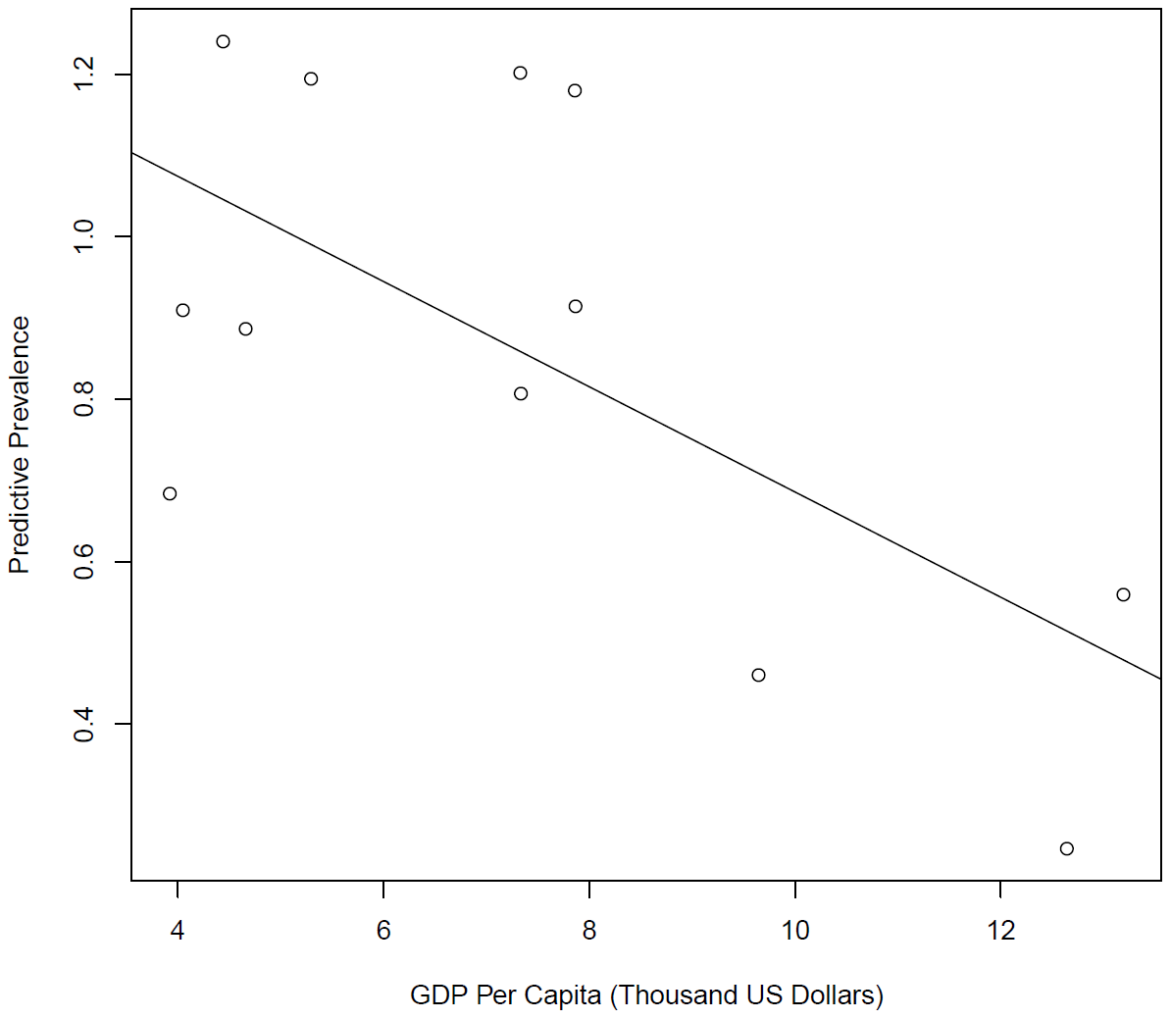
Bayesian meta-regression indicated GDP per capita negatively associated with the prevalence.
