# Supplementary material for: A Bayesian Meta-Analysis on Prevalence of Hepatitis B Virus Infection among Chinese Volunteer Blood Donors
Source: PLoS One. 2013 Nov 13;8(11):e79203. doi: 10.1371/journal.pone.0079203 (PMC3827339; doi:10.1371/journal.pone.0079203)
Supplement: Appendix S1 — The statistical models of Bayesian meta-analysis and meta-regression. (DOC) [file pone.0079203.s001.doc]

**Appendix S1:** The statistical models of Bayesian meta-analysis and meta-regression.

All Bayesian models were fitted using WinBugs version 1.4 (MRC Biostatistics Unit, Cambridge, UK).

**Model using for pooling results:**

Prior distribution at Level 1:

Let r_i_ be the cases of HBV infection in study i,

$$\begin{matrix} r_{i}\sim Bin\left(p_{i}\right.,\left.n_{i}\right) \\ p_{i}\sim Beta\left(a\right.,\left.b\right) \end{matrix}$$

Where p_i_ is the prevalence of HBV infection in study i

Prior distribution at Level 2:

$$\begin{matrix} a\sim unif\left(0\right.,\left.20000\right) \\ b\sim unif\left(0\right.,\left.20000\right) \\ mu=\frac{a}{a+b} \end{matrix}$$

**Model used for checking results:**

Prior distribution at Level 1:

Let r_i_ be the cases of HBV infection in study i,

$$\begin{matrix} r_{i}\sim Bin\left(p_{i}\right.,\left.n_{i}\right) \\ \log it\left(p_{i}\right)=mu_{i} \\ mu_{i}\sim Norm\left(mu\right.,\left.a.tau\right) \end{matrix}$$

Where pi is the prevalence of HBV infection in study i

Prior distribution at Level 2:

$$\begin{matrix} mu.a\sim Norm\left(0\right.,\left.1\right) \\ tau\sim Gamma\left(0.001\right.,\left.0.001\right) \\ result=\frac{\exp\left(mu.a\right)}{1+\exp\left(mu.a\right)} \end{matrix}$$
